# Noradrenaline enhances the excitatory effects of dopamine on medial prefrontal cortex pyramidal neurons in rats

**DOI:** 10.1002/npr2.12135

**Published:** 2020-09-08

**Authors:** Fumiya Shinohara, Saya Arakaki, Taiju Amano, Masabumi Minami, Katsuyuki Kaneda

**Affiliations:** ^1^ Department of Pharmacology Graduate School of Pharmaceutical Sciences Hokkaido University Sapporo Japan; ^2^ Laboratory of Molecular Pharmacology Institute of Medical, Pharmaceutical and Health Sciences Kanazawa University Kanazawa Japan

**Keywords:** cocaine, dopamine, medial prefrontal cortex, noradrenaline, stress

## Abstract

**Aim:**

Our previous studies showed that exposure to acute restraint stress enhanced cocaine‐induced conditioned place preference (cocaine‐CPP) and suggested the possibility that co‐activation of adrenergic transmission boosts the increase in medial prefrontal cortex (mPFC) neuronal activity by the activation of dopaminergic transmission. To examine this possibility, the effects of the co‐treatment with dopamine (DA) and noradrenaline (NA) on mPFC neurons were compared with those of treatment with DA alone using whole‐cell patch‐clamp recordings.

**Methods:**

The effects of DA alone and a mixture of DA and NA on the membrane potentials and spontaneous excitatory postsynaptic currents (sEPSCs) were examined by electrophysiological recordings of mPFC pyramidal neurons in brain slices of male Sprague Dawley rats. Extracellular DA and NA levels in the mPFC during and after restraint stress exposure were also examined by in vivo microdialysis.

**Results:**

Dopamine significantly produced depolarizing effects on mPFC neurons and tended to increase sEPSC frequency. Co‐administration of NA with DA produced stronger depolarizing effects and significantly increased sEPSC frequency. The findings suggest that the additional depolarizing effect of NA on DA‐responsive neurons, rather than the excitation of DA‐nonresponsive neurons by NA, contributes to the stronger effect of co‐treatment of NA with DA.

**Conclusion:**

The present study suggests that NA released by restraint stress exposure cooperates with DA to stimulate DA‐responsive neurons in the mPFC, thereby causing the stress‐induced enhancement of cocaine‐CPP.

## Abstract 2

Our previous studies showed that exposure to acute restraint stress enhanced cocaine‐induced conditioned place preference (cocaine‐CPP). The present study revealed that noradrenaline enhanced the excitatory effects of dopamine on medial prefrontal cortex (mPFC) pyramidal neurons in rats. Noradrenaline released by restraint stress exposure may cooperate with dopamine to stimulate dopamine‐responsive neurons in the mPFC, thereby causing the stress‐induced enhancement of cocaine‐CPP.

## Graphical abstract

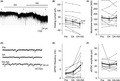

## 1 INTRODUCTION

Craving for drugs of abuse (eg, cocaine) is induced or potentiated by stress in humans and rodents.^1^, ^2^ Therefore, comprehension of the neural mechanisms of stress‐induced or stress‐potentiated cocaine craving is important for the development of effective treatments of cocaine use disorder. Several previous studies showed that acute stress induces or potentiates cocaine craving in rodents;^3^, ^4^, ^5^ we also reported that a 30‐minute restraint stress immediately before the expression (postconditioning) session of the conditioned place preference (CPP) test enhanced cocaine‐induced CPP (cocaine‐CPP) in rats.^6^ Additionally, we showed that blockade of dopamine (DA) D_1_ receptors in the rat medial prefrontal cortex (mPFC) suppressed both cocaine‐CPP^7^ and stress‐induced enhancement of cocaine‐CPP.^6^ Moreover, we have recently found that blockade of noradrenaline (NA) α_1_ receptors in the mouse mPFC suppressed the stress‐induced enhancement of cocaine‐CPP, but not cocaine‐CPP itself.^8^ These findings suggest that dopaminergic transmission in the mPFC is necessary for cocaine‐CPP, while noradrenergic transmission in the mPFC is not required for cocaine‐CPP, but can enhance this preference. Co‐activation of α_1_ receptors may enhance the increase in mPFC neuronal activity by means of D_1_ receptor activation. To examine this possibility, a whole‐cell patch‐clamp technique was used to investigate the excitatory effects of co‐treatment with DA and NA on the mPFC neurons, compared to the effects of treatment with DA alone.

## 2 MATERIALS AND METHODS

### 2.1 Animals

Twenty‐eight male Sprague Dawley rats (weighing 170‐240 g) from Japan SLC (Hamamatsu, Japan) were maintained in a temperature‐controlled (22 ± 1°C) room under a 12‐hour light/dark cycle with food and water available ad libitum. All experiments were conducted with the approval of the Hokkaido University Institutional Animal Care and Use Committee. All efforts were made to minimize the number and suffering of animals used in the experiments.

### 2.2 Drugs

Cocaine hydrochloride (Takeda Pharmaceutical) was dissolved in saline. DA hydrochloride and NA hydrochloride were purchased from Sigma‐Aldrich. Stock solutions of DA and NA were dissolved in water at concentrations of 20 and 10 mmol/L, respectively, and diluted to final concentrations (DA, 20 μmol/L; NA, 10 μmol/L) with recording solution prior to bath application.

### 2.3 Slice preparation for electrophysiology

The animals were euthanized under isoflurane anesthesia (2%) by transcardial perfusion with ice‐cold cutting solution (in mM: 92 N‐methyl‐D‐glucamine, 2.5 KCl, 30 NaHCO_3_, 1.25 NaH_2_PO_4_, 25 glucose, 5 sodium ascorbate, 0.5 CaCl_2_, 20 HEPES, 10 MgSO_4_, 2 thiourea, 3 sodium pyruvate, and 12 N‐acetyl‐L‐cysteine, oxygenated with 95% O_2_/5% CO_2_ adjusted to pH 7.4 ± 0.1 with HCl). The brains were quickly removed and coronal slices (250 μm thick) containing the mPFC were prepared in ice‐cold cutting solution with a vibratome (VT1200S; Leica Microsystems GmbH). The slices were incubated for 15 minutes at 32‐34°C in the cutting solution and subsequently kept in the recording solution (in mM: 119 NaCl, 2.5 KCl, 24 NaHCO_3_, 1.25 NaH_2_PO_4_, 12.5 glucose, 2 CaCl_2_, and 2 MgSO_4_, oxygenated with 95% O_2_/5% CO_2_) at room temperature for at least 1 hour. The slices were transferred to a submerged recording chamber on an upright microscope (BX50WI; Olympus) and continuously superfused with recording solution at 35 ± 1°C saturated with 95% O_2_/5% CO_2_ at a flow rate of 2 mL/min. Glass pipettes were pulled from thin‐walled borosilicate glass capillaries with a micropipette puller (Model P‐1000IVF; Sutter Instrument). Tip resistance was 4.0‐9.0 MΩ when glass pipettes were filled with internal solution (in mM: 150 KOH, 2 MgCl_2_, 10 KCl, 0.2 EGTA, 2 Na_2_‐ATP, 0.3 Na_2_‐GTP, 10 HEPES, and 0.1 spermine, adjusted to pH 7.3 ± 0.1 with gluconic acid).

### 2.4 Measurement of holding currents and spontaneous excitatory postsynaptic currents

Layer V pyramidal neurons in the mPFC prelimbic area were identified by their large size and pyramidal shape at a depth of 700‐900 μm from the cortical surface. All recorded pyramidal neurons exhibited spike frequency adaptation as reported previously.^9^ The membrane potential was voltage‐clamped at –70 mV, and the holding currents and spontaneous excitatory postsynaptic currents (sEPSCs) were recorded. After a stable holding current had been observed, DA was bath‐applied for 5 minutes, followed by the bath application of a cocktail of DA and NA (DA + NA) for 5 minutes. Holding currents and sEPSCs were measured 0‐1 minute before and 4‐5 minutes after the start of the application of DA alone, and 4‐5 minutes after the start of the application of DA + NA. To examine the effects of NA alone on the membrane potential, holding currents were measured 0‐1 minute before and 4‐5 minutes after the start of the application of NA alone. The averaged holding current value was obtained by averaging holding currents during 10‐s windows, which were set every 30 seconds. All data were acquired with a Multiclamp 700B amplifier and pClamp10 software (Molecular Devices). Data from neurons whose resting membrane potential was more positive than –50 mV or in which the action potential did not overshoot were excluded from statistical analyses. Access resistance was monitored by injecting a voltage pulse (–5 mV, 50 ms) at 30‐s intervals. Data from neurons for which access resistance changed more than 20% during recordings were excluded from statistical analyses. The frequency and amplitude of sEPSCs were analyzed using the Mini Analysis Program (Synaptosoft, Fort Lee, NJ, USA).

### 2.5 In vivo microdialysis

Under isoflurane (2.5%) anesthesia, a microdialysis guide cannula (o.d., 0.5 mm; AG‐4; Eicom, Kyoto, Japan) was implanted unilaterally 1.0 mm above the mPFC (2.7 mm rostral, 0.7 mm lateral, and 3.0 mm ventral to the bregma). The animals were individually housed in cages for a recovery period of 5‐7 days. In vivo microdialysis was conducted in unanesthetized freely moving rats. A microdialysis probe (dialysis membrane, 1000 kDa molecular weight cutoff polyethylene membrane; length, 1.0 mm; o.d., 0.22 mm; A‐I‐4‐01; Eicom) was inserted through the guide cannula and continuously perfused with Ringer's solution (147 mmol/L NaCl, 4 mmol/L KCl, and 2.3 mmol/L CaCl_2_) at a flow rate of 1 μL/min. The rats were placed in a Plexiglas chamber (width × depth × height: 30 × 30 × 35 cm). After the extracellular DA and NA levels had been stabilized, three 15‐min dialysate fractions were collected as baseline samples. During the last approximately 3 minutes of the subsequent (ie, fourth) 15‐min fraction collection period, the procedure for animal restraint was carried out. Animals were restrained by wrapping with four pieces of thin plastic sheets with both ends pasted with adhesive tape and overwrapping with a towel. Two 15‐min fractions were collected during the 30‐min stress exposure period. Following release of the animals from the restraint, eight 15‐min fractions were collected. Each dialysate sample was separated on a liquid chromatography column (Eicompak CAX, 2.0 mm i.d. ×200 mm; Eicom) at 35°C using 0.1 mol/L ammonium acetate buffer (pH 6.0) containing 0.05 mol/L sodium sulfate, 50 mg/L ethylenediaminetetraacetic acid, and 30% methanol at a constant flow rate of 0.25 mL/min. DA and NA contents were measured using an electrochemical detector (HTEC‐500; Eicom), with a working electrode set at +450 mV versus an Ag/AgCl reference electrode. Chromatogram peaks were analyzed using the PowerChrom data‐recording system (Eicom). Data are expressed as a percent of baseline, which was calculated as the average of the three baseline samples. The criteria to exclude data from statistical analyses were baseline values less than 0.025 and/or 0.1 pg/sample for DA and NA, respectively, an unstable baseline level (defined as >30% difference among the three baseline samples), or a bursting increase in serotonin level likely due to microhemorrhage. In this study, one animal was excluded due to an unstable baseline level. Histological analyses were performed after in vivo microdialysis. Rats were decapitated, and their brains were rapidly removed and frozen in powdered dry ice. Coronal sections (50 µm) including the mPFC were prepared using a cryostat, thaw‐mounted onto slides, stained with thionine, and examined under a microscope (40×). The criteria to exclude data from statistical analyses were misplaced microdialysis probe or extensive tissue damage. In this study, two animals were excluded due to a misplaced microdialysis probe.

### 2.6 Statistical analyses

Statistical analyses were performed using GraphPad Prism v. 6.00 (GraphPad Software). Data from the electrophysiological experiments were analyzed using a one‐way repeated‐measures analysis of variance (ANOVA), followed by Tukey's *post hoc* test. Data from the microdialysis experiments were analyzed using one‐way repeated‐measures ANOVA, followed by Dunnett's multiple comparison test. Differences with *P* < .05 were considered significant. Law data of the electrophysiological and in vivo microdialysis experiments were available in Appendix S1 and S2, respectively.

## 3 RESULTS

### 3.1 Effects of DA alone and a mixture of DA and NA on the excitability of mPFC layer V pyramidal neurons

**FIGURE 1:**
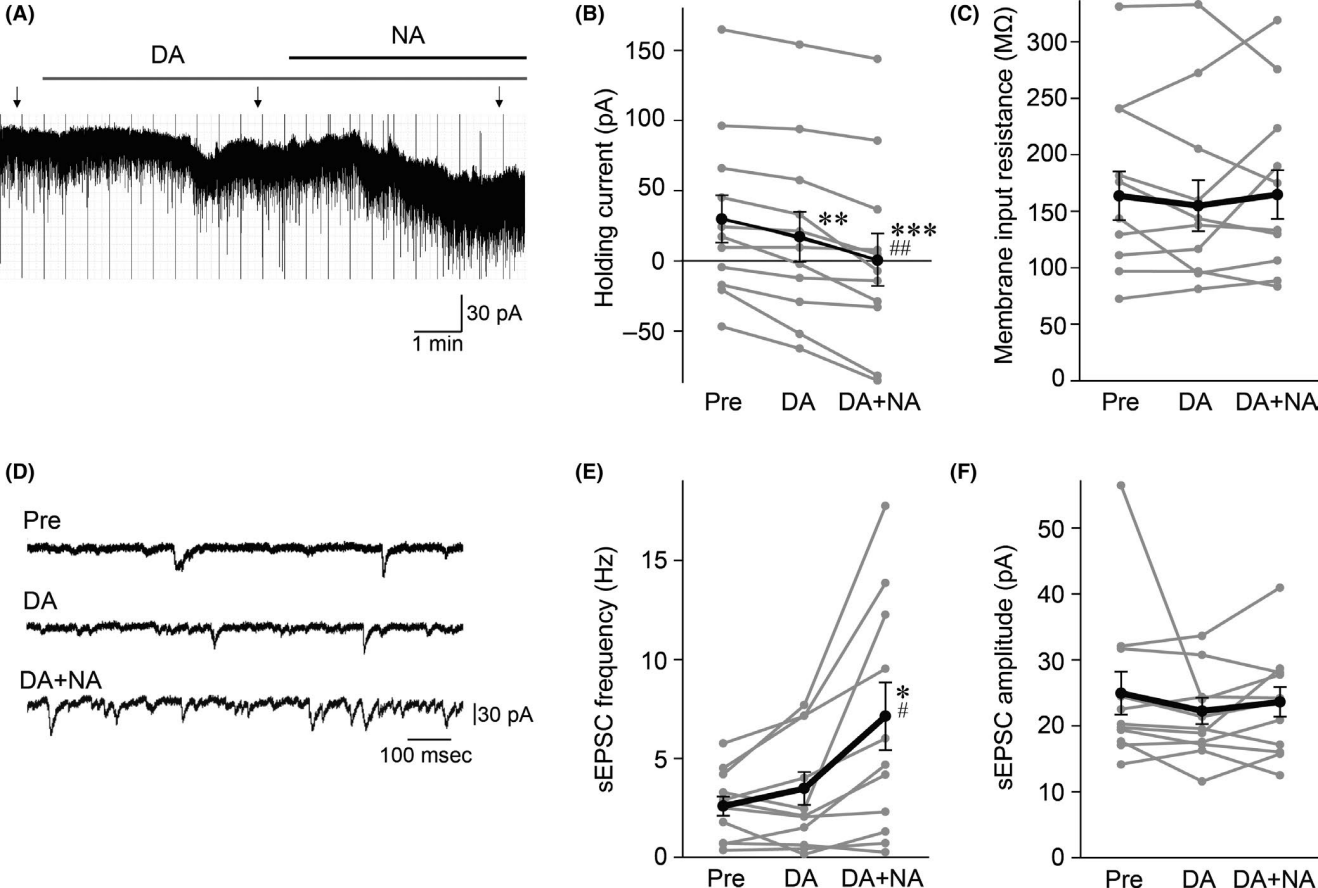
Excitatory effects of dopamine (DA) alone, as well as a mixture of DA and noradrenaline (NA; DA + NA), on medial prefrontal cortex (mPFC) layer V pyramidal neurons. A, Representative trace of holding currents. B and C, Effects of DA and DA + NA on holding current (B) and membrane input resistance (C) (n = 12 from 11 rats). ^**^ *P* < .01, ^***^ *P* < .001 vs. Pre, ^##^ *P* < .01 vs DA (one‐way repeated‐measures ANOVA, followed by Tukey's *post hoc* test). D, Expansion traces from points indicated by arrows in panel A showing spontaneous excitatory postsynaptic currents (sEPSCs) obtained before (Pre, top) and after (DA, middle) application of DA alone, as well as after application of a mixture of DA and NA (DA + NA, bottom). E and F, Effects of DA and DA + NA on sEPSC frequency (E) and amplitude (F) (n = 12 from 11 rats). ^*^ *P* < .05 vs Pre, ^#^ *P* < .05 vs DA (one‐way repeated‐measures ANOVA, followed by Tukey's *post hoc* test). B, C, E, and F, Data shown by black symbols and lines represent means ± standard errors of the mean (SEM); gray symbols and lines indicate data from individual cells

Bath application of DA (20 μmol/L) led to significant change in holding currents, from 29.9 ± 16.8 pA to 17.2 ± 17.8 pA (n = 12 neurons from 11 rats, *F*_1.197, 13.17_ = 26.14, *P* = .0001, one‐way repeated‐measures ANOVA; Pre vs. DA, *P* = .0019, *post hoc* Tukey's test, Figure 1A,B). Input resistance was not changed by DA (*F*_1.313, 14.44_ = 0.4832, *P* = .5485, one‐way repeated‐measures ANOVA; Pre: 163.7 ± 21.6 MΩ vs DA: 154.9 ± 22.5 MΩ, Figure 1C). The frequency and amplitude of sEPSCs (Figure 1D–F) were not significantly changed by DA (frequency, *F*_1.168, 12.85_ = 10.17, *P* = .0057, one‐way repeated‐measures ANOVA; Pre: 2.6 ± 0.5 Hz vs. DA: 3.5 ± 0.8 Hz, *P* = .2956, *post hoc* Tukey's test; amplitude, *F*_1.318, 14.50_ = 0.59, *P* = .4986, one‐way repeated‐measures ANOVA; Pre: 24.9 ± 3.3 pA vs DA: 22.3 ± 2.0 pA).

Additive application of NA (10 μmol/L) in the presence of DA (20 μmol/L) (DA + NA) produced an additional change in the holding current (0.7 ± 18.7 pA, Pre vs. DA + NA, *P* = .0006, DA vs DA + NA, *P* = .0016, *post hoc* Tukey's test, Figure 1A,B). DA + NA did not change input resistance (164.7 ± 21.6 MΩ, Figure 1C). DA + NA significantly increased sEPSC frequency 7.1 ± 1.7 Hz), compared to Pre and DA (Pre vs. DA + NA, *P* = .0203, DA vs. DA + NA, *P* = .0138, *post hoc* Tukey's test, Figure 1D,E), whereas DA + NA did not change sEPSC amplitude (23.6 ± 2.3 pA, Figure 1D–F).

Neurons that showed changes in holding currents of more than 10 pA in the negative or positive direction were designated as excitatory or inhibitory responders, respectively. Table 1 shows that seven of the 12 neurons were excitatory responders to DA, while five were nonresponders. No inhibitory responders were among the 12 neurons examined. Furthermore, six of the seven DA‐responsive neurons showed additional excitation in response to NA, whereas one did not. Conversely, two of the five DA‐nonresponsive neurons were excited by NA, while three were not. Using the separate cohort of mPFC neurons, we examined the effects of NA alone. Ten of the 11 neurons were excitatory responders to NA, while one was a nonresponder.

**Table 1:** Changes in holding current following application of DA, DA + NA, and NA

Neuron #	DA	DA + NA	Neuron #	NA
	Δ holding current (pA)	Δ holding current (pA)		Δ holding current (pA)
1	+	+	1	+
	(−19.6)	(−26.6)		(−92.7)
2	+	+	2	+
	(−12.1)	(−40.4)		(−11.0)
3	±	±	3	+
	(−7.8)	(−1.8)		(−14.2)
4	±	+	4	±
	(−8.5)	(−21.1)		(−7.6)
5	+	+	5	+
	(−31.3)	(−29.9)		(−12.5)
6	±	+	6	+
	(−3.2)	(−16.5)		(−79.4)
7	±	±	7	+
	(0.2)	(−1.7)		(−13.7)
8	+	±	8	+
	(−12.3)	(−3.8)		(−18.8)
9	+	+	9	+
	(−12.8)	(−10.3)		(−40.3)
10	±	±	10	+
	(−2.6)	(−8.0)		(−11.2)
11	+	+	11	+
	(−15.8)	(−22.8)		(−26.7)
12	+	+		
	(−25.8)	(−15.9)		

Note

+, excitatory responder (change in holding current in the negative direction by more than 10 pA); and ±, nonresponder (change in holding current by <10 pA). Numerical values in parentheses indicate Δ holding currents (pA).

### 3.2 Acute restraint stress increased extracellular DA and NA levels in the mPFC

**FIGURE 2:**
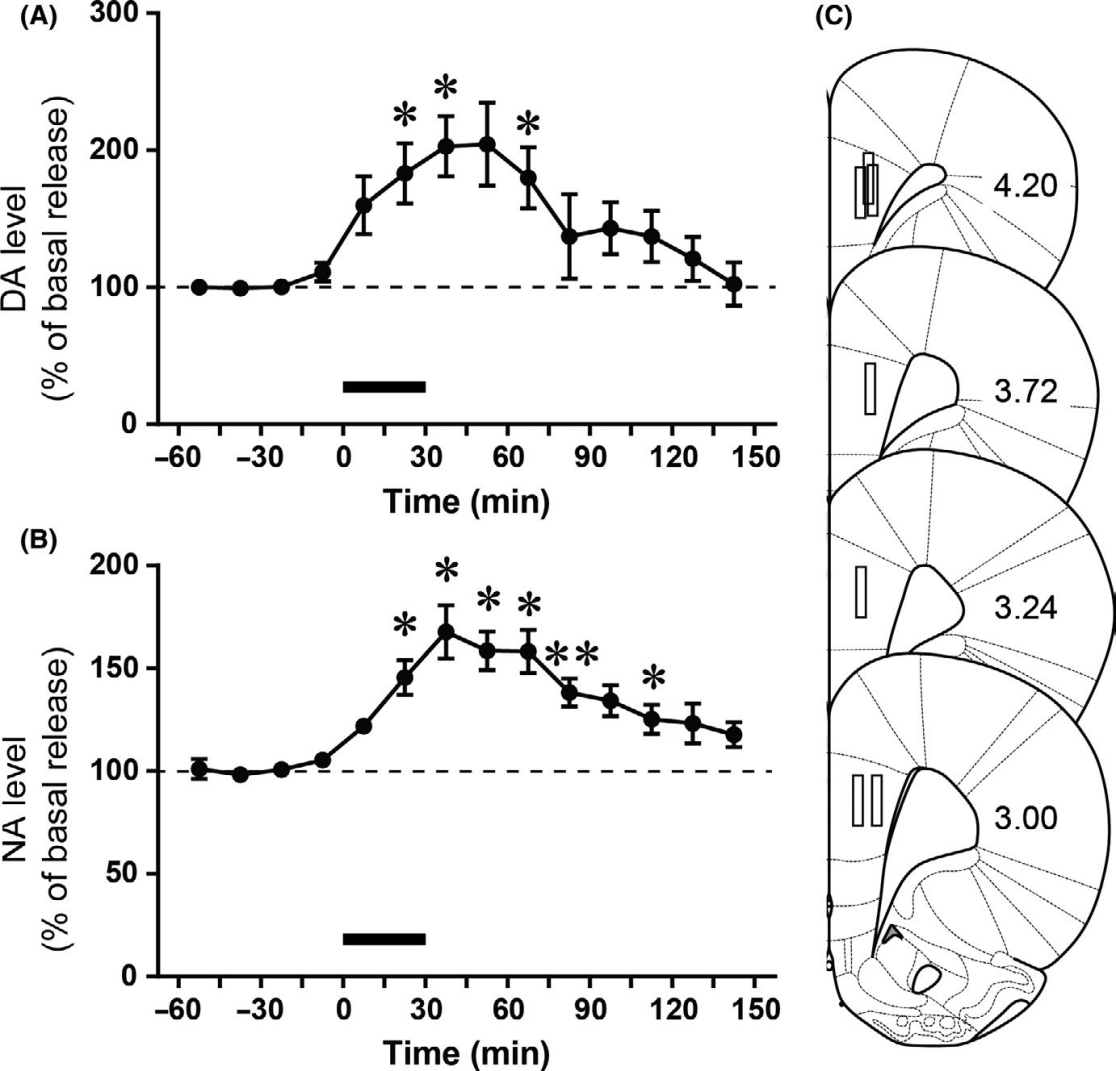
A and B, Acute restraint stress‐induced increases in extracellular DA (A) and NA (B) levels in the rat mPFC. Animals were exposed to restraint stress for 30 min started at time 0 (shown by horizontal bar in graph). Data are expressed as means ± SEM ^*^ *P* < .05, ^**^ *P* < .01 vs. last baseline sample (one‐way repeated‐measures ANOVA, followed by Dunnett's multiple comparison test)

Exposure to acute restraint stress led to elevated extracellular DA levels in the mPFC (Figure 2A). One‐way repeated‐measures ANOVA demonstrated a significant effect of the stress exposure (*F*_2.282, 13.69_ = 8.476, *P* = .0032, n = 7). A significant increase in the DA level, compared with the last baseline sample (100.5 ± 4.21%), was observed during and after the stress exposure (15‐30 min: 183.1 ± 22.0%, *P* = .0331; 30‐45 min: 202.8 ± 22.1%, *P* = .0125; and 60‐75 min: 179.8 ± 22.4%, *P* = .0440, Dunnett's multiple comparison test). Acute restraint stress also increased extracellular NA levels in the mPFC (Figure 2B). One‐way repeated‐measures ANOVA demonstrated a significant effect of the stress exposure (*F*_2.363, 14.18_ = 14.83, *P* = .0002, n = 7). A significant increase in the NA level, compared with the last baseline sample (100.8 ± 3.27%), was observed during and after the stress exposure (15‐30 minutes: 145.6 ± 8.54%, *P* = .0416; 30‐45 minutes: 167.7 ± 13.1%, *P* = .0293; 45‐60 minutes: 158.6 ± 9.44%, *P* = .0143; 60‐75 minutes: 158.2 ± 10.6%, *P* = .0234; 75‐90 minutes: 138.3 ± 6.86%, *P* = .0081; and 105‐120 minutes: 125.3 ± 7.00%, *P* = .0334, Dunnett's multiple comparison test).

## 4 DISCUSSION

Whole‐cell patch‐clamp electrophysiology showed that DA significantly changed the holding current in the negative direction, indicating a depolarizing effect of DA on mPFC pyramidal neurons. While DA tended to increase the frequency of sEPSCs, this difference was not statistically significant. Co‐administration of NA with DA produced stronger effects on the membrane potential and significantly increased the frequency of sEPSCs. Focusing on the effects of DA alone and co‐treatment with DA and NA on individual neurons, seven of the 12 neurons were excitatory responders to DA, while five were nonresponders. No inhibitory responders were found among the 12 neurons examined. Furthermore, six of the seven DA‐responsive neurons showed additional excitation in response to NA, whereas one did not. In contrast, two of the five DA‐nonresponsive neurons were excited by NA, while three were not. These results suggest that the additional depolarizing effect of NA on DA‐responsive neurons, rather than the excitation of DA‐nonresponsive neurons by NA, contributes to the stronger effect of co‐treatment with DA and NA on the membrane potentials of mPFC pyramidal neurons. mPFC neurons from the separate cohort of animals were utilized to examine the effects of NA alone. Ten of the 11 neurons were excitatory responders to NA, while one was a nonresponder. These results show that excitatory effect of NA on mPFC neurons does not need the pretreatment with DA.

Activation of D_1_ receptors has been reported to depolarize mPFC pyramidal neurons via inhibition of G protein‐dependent inwardly rectifying potassium currents.^10^ Furthermore, NA has been shown to enhance excitability of mPFC pyramidal neurons by increasing hyperpolarization‐activated cyclic nucleotide‐gated (*I*_h_) current ^11^ and decreasing TREK‐2 like channel currents.^12^ These channels may be involved in the depolarizing effects of DA and NA observed in this study. We previously demonstrated that NA shows the depolarizing effects which lead to the induction of action potentials in mouse mPFC pyramidal neurons.^8^ Thus, it is likely that the depolarizing effects of DA and NA increase firing rates in rat mPFC pyramidal neurons.

We found that co‐application of DA and NA significantly increased the frequency of sEPSCs in mPFC neurons, whereas application of DA alone led to a slight increase in sEPSC frequency. These results are consistent with the findings of previous studies, in which DA and NA increased the sEPSC frequencies in rat mPFC layer V pyramidal neurons by 1.7‐ and 4.5‐fold, respectively.^13^ Luo et al reported that activation of α_1_ receptors facilitates excitatory synaptic transmission in rat mPFC pyramidal neurons via both pre‐ and postsynaptic PKC‐dependent mechanisms.^14^ We recently reported that NA increases sEPSC frequency in mouse mPFC layer V pyramidal neurons. This increase is induced, at least in part, by postsynaptic action of NA: Specifically, NA excites mPFC pyramidal neurons, which then increase glutamate release in synaptic terminals.^8^

Consistent with the findings of a previous study,^15^ restraint stress increased extracellular DA and NA levels in the mPFC with similar time courses. Extracellular levels of these neurotransmitters significantly increased during the latter half of 30‐minute restraint stress exposure (15‐30 minutes) and the 15‐minute period after stress (30‐45 minutes), which correspond to the postconditioning test session of the CPP test in our previous study.^6^ We previously reported that blockade of D_1_ receptors in the mPFC during the postconditioning test session suppressed cocaine‐CPP, suggesting an important role for dopaminergic transmission in the expression of cocaine‐CPP. Elevated levels of DA in the mPFC due to restraint stress may contribute to stress‐induced enhancement of cocaine‐CPP by boosting dopaminergic transmission during the postconditioning test session. We have found that blockade of α_1_ receptors in the mouse mPFC suppresses the stress‐induced enhancement of cocaine‐CPP, but not cocaine‐CPP itself; this finding suggest that noradrenergic transmission is not required for cocaine‐CPP, but can enhance this preference. Electrophysiological analyses in the present study suggested that NA enhances the excitability of mPFC pyramidal neurons by an additional depolarizing effect on DA‐responsive neurons, rather than by excitation of DA‐nonresponsive neurons. These findings suggest that DA and NA released by restraint stress exposure strongly excite DA‐responsive neurons by co‐activation of D_1_ and α_1_ receptors in mPFC pyramidal neurons, thereby causing stress‐induced enhancement of cocaine‐CPP.

## CONFLICT OF INTEREST

The authors declare no conflict of interests.

## AUTHOR CONTRIBUTIONS

KK, FS, and MM designed the experiments and prepared the manuscript. FS performed the electrophysiological experiments. SA performed the in vivo microdialysis experiments. KK, FS, and TA analyzed the data.

## ANIMAL STUDIES

All the experimental procedures were approved by the Hokkaido University Institutional Animal Care and Use Committee.

## Supporting information

App S1

App S2

## Data Availability

The data that supports the findings of this study are available in the supplementary material of this article.
